# Personal

**Published:** 1888-11

**Authors:** 


### Personal.

Mr. F. C. M. Lautz, President of the Queen City Cyclorama Co., and his coädjutors in the Board of Directors, have done a praiseworthy act in erecting a permanent place of exhibition for "Jerusalem on the Day of Crucifixion," on Music Hall Grounds. Here the student of biblical literature and of heroic art, as well as the curious and uninstructed spectator, may spend an hour or two with satisfaction and profit. Such enterprise is to be commended by every person who would encourage a higher standard of morality, religious thought and education ; and every citizen of Buffalo who has the best interests of the city at heart, should encourage the establishment of such places of entertainment within its borders. It is in this spirit that we have stepped aside from the usual policy of the Journal, and given place to a word of commendation to an object outside of strictly professional interests.

Dr. J. Henry Carstens was, at the annual meeting held October 1, 1888, elected President of the Detroit Medical and Library Association for the ensuing year. This prosperous society includes in its membership some of the most influential and celebrated physicians in our neighboring city. The retiring President, Dr. C. G. Jennings, entertained the Society in his hospitable home after the adjournment of the annual meeting.

Dr. Thomas Opie was, at the meeting held October 9, 1888, elected President of the Maryland Obstetrical and Gynecological

Society for the ensuing year. This is a fitting recognition of the ability of one of the most accomplished obstetricians in the Monumental City, and we feel confident that the continued prosperity of this useful Society is assured under President Opie's administration.

---

Mr. Victor Horsley, the distinguished English surgeon, who is especially noted for his studies in cerebral localization, paid Buffalo a brief visit as the guest of Dr. Park, during the early part of last month. At a reception given in his honor by Dr. Park on the evening of October 10th, the profession of this city had an opportunity of meeting Mr. Horsley, an entertainment that will long be remembered as enjoyable and attractive.

---

Dr. Joseph Price, of Philadelphia, recently paid a flying visit to Buffalo, on his way from Western Ohio, whither he had been to perform ovariotomy, to Niagara Falls. The Journal hopes his next visit will not be as brief as the last, that an opportunity may be afforded to show the distinguished Philadelphian more of the Queen City of the Lakes. Dr. Price spoke in pleasant terms of what he saw, and was particularly interested in the crematory, pronouncing it the most artistic and best built structure of the kind he had yet seen.

---

Dr. Wm. Osler, Professor of Clinical Medicine in the University of Pennsylvania, has been elected Professor of the Practice of Medicine in Johns Hopkins University. The liberal endowment of this institution enables it to seek and obtain the very best teaching talent the country affords, and will make it a model university if it always exercises the same discretion that led to a choice in this instance. Prof. Osler will continue to fill his present chair until May next, when he will remove to Baltimore.

---

Dr. William T. Lusk was, at its recent annual meeting, elected President of the New York State Medical Association.

